# Case Report: Recurrent pelvic gestational choriocarcinoma arising from an occult complete mole unmasked by short tandem repeat genotyping

**DOI:** 10.3389/fonc.2026.1854233

**Published:** 2026-08-04

**Authors:** He Qiu, Tingting Chen, Fenghua Ma, Weiguo Hu, Xin Lu, Jiahui Ma, Yan Du, Xiaoni Yue

**Affiliations:** 1 Clinical Research Unit, Obstetrics and Gynecology Hospital of Fudan University, Shanghai, China; 2 Shanghai Key Lab of Reproduction and Development, Shanghai, China; 3 Shanghai Key Lab of Female Reproductive Endocrine Related Diseases, Shanghai, China; 4 Department of Pathology, Obstetrics and Gynecology Hospital of Fudan University, Shanghai, China; 5 Department of Radiology, Obstetrics and Gynecology Hospital of Fudan University, Shanghai, China; 6 Department of Gynecology Oncology, Obstetrics and Gynecology Hospital of Fudan University, Shanghai, China

**Keywords:** complete hydatidiform mole, extrauterine choriocarcinoma, gestational trophoblastic neoplasia, short tandem repeat genotyping, surgical resection

## Abstract

**Background:**

Pelvic gestational choriocarcinoma (GCC) is very rare. It may mimic ectopic pregnancy because of the elevated human chorionic gonadotropin (hCG) levels and absence of typical uterine lesions. After choriocarcinoma is identified, accurate determination of tumor origin is critical for prognostic risk stratification and treatment selection.

**Case presentation:**

A 29-year-old woman presented with irregular vaginal bleeding and markedly rising hCG levels, with imaging revealing a pelvic mass, and was initially diagnosed with ectopic pregnancy. Emergency laparoscopy and pathological review confirmed choriocarcinoma, but the resected tissue was insufficient for short tandem repeat (STR) genotyping to determine the tumor origin. Based on her pregnancy history and age, she was empirically treated for presumed GCC and achieved complete remission after chemotherapy. Approximately 3 years later, she developed an isolated recurrence at the same location. She then underwent three-dimensional laparoscopic resection, which enabled STR genotyping. The analysis revealed a homozygous androgenetic diploid pattern, definitively confirming gestational origin and establishing the causative gestation as a complete hydatidiform mole, thereby revising the prognostic score from 9 to 8. Postoperatively, the patient received five cycles of chemotherapy, serum hCG normalized, and she remains disease-free at 25 months of follow-up.

**Conclusions:**

The case underscores two key messages. First, STR genotyping is highly valuable for accurate etiological classification and risk stratification in extrauterine choriocarcinoma. Second, surgical resection of an isolated chemoresistant or recurrent lesion, even at elevated hCG levels, can provide essential tissue for molecular diagnosis and facilitate rapid biochemical remission.

## 1 Introduction

Choriocarcinoma is a trophoblastic malignancy subtyped as gestational (GCC) or non-gestational choriocarcinoma (NGCC). GCC usually follows a molar pregnancy, abortion, or term delivery, responds well to chemotherapy, and most commonly arises in the uterus (1). NGCC arises from germ or somatic cells, carries a poorer prognosis, and is typically chemoresistant (2). Extrauterine GCC, though rare, can involve ovaries, fallopian tubes, vagina, and other abdominopelvic sites that may mimic ectopic pregnancy and complicate diagnosis (3, 4). Even when choriocarcinoma is confirmed, gestational versus non-gestational origin and the causative pregnancy cannot be reliably determined based solely on age, pregnancy history, menstrual status, tumor location, and histomorphology (5, 6). Short tandem repeat (STR) genotyping is an effective ancillary tool in the diagnosis of gestational trophoblastic neoplasia (GTN), as it compares tumor DNA with maternal and paternal allele patterns to determine the parental origin and ploidy (7).

We report a pelvic GCC that recurred at the same location after complete remission. STR genotyping of the recurrent lesion identified androgenetic diploidy indicative of a complete hydatidiform mole (CHM) as the causative gestation, thereby refining the International Federation of Gynecology and Obstetrics (FIGO) risk scoring and informing subsequent management.

## 2 Case description

**Figure 1:**
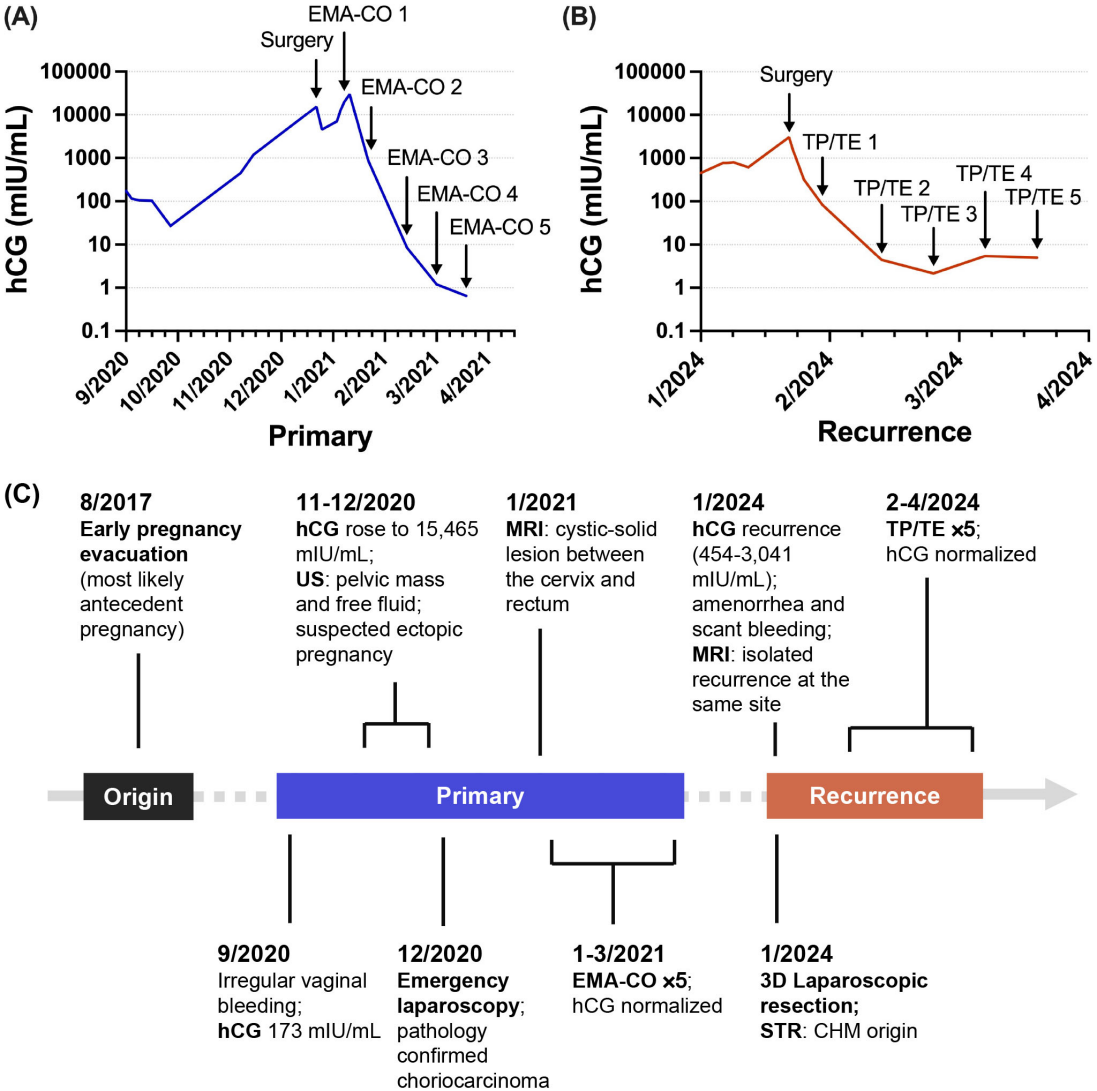
Serum hCG levels in primary and recurrent settings and summary timeline of key clinical events. **(A)** Serum hCG levels (logarithmic scale) during the primary diagnosis and treatment phase. **(B)** Serum hCG levels (logarithmic scale) during the recurrent phase. Arrows indicate the timing of surgery and chemotherapy. **(C)** Summary timeline of key clinical events.

A 29-year-old woman (gravida 2, para 1) presented in September 2020 with 1 month of irregular vaginal bleeding. Her obstetric history included a term vaginal delivery in 2016 and a surgical evacuation for an early pregnancy in August 2017; chorionic villi and decidua were noted, but no histopathologic evaluation was performed after the evacuation. After presentation, her initial serum human chorionic gonadotropin (hCG) was 173 mIU/mL (**Figure 1A**), and ultrasonography showed an endometrial thickness of 10 mm and a 6.0 × 4.0 cm left ovarian cyst. Over the following 2 months, serum hCG fluctuated between 27 and 447 mIU/mL (**Figure 1A**), and no intrauterine gestational sac was visualized despite three episodes of unprotected intercourse. From November 2020, hCG rose rapidly, reaching 15,465 mIU/mL by December (**Figure 1A**). Repeat ultrasound revealed endometrial heterogeneity, a mixed-echo posterior uterine mass measuring 9.6 × 3.3 cm adjacent to the uterine corpus, and pelvic free fluid measuring 8.0 × 3.3 cm, raising suspicion of ectopic pregnancy.

Emergency laparoscopy was performed at a local hospital, which comprised excision of a right pelvic peritoneal lesion, left ovarian cystectomy, and dilation and curettage. The surgical specimens were subsequently reviewed at our institution. The postoperative pathology confirmed choriocarcinoma showing atypical trophoblasts with high proliferative activity and no villous structures; immunohistochemistry was positive for hCG and hydroxy-delta-5-steroid dehydrogenase, 3 beta- and steroid delta-isomerase 1 (HSD3B1), with a Ki-67 index of 80%. Microscopic examination revealed only scant viable trophoblastic tumor cells against a background of blood clot in the sample. Although this allowed histopathological and immunohistochemical diagnosis, the tumor component was insufficient for reliable STR analysis. Therefore, STR genotyping was not performed at this stage.

**Figure 2:**
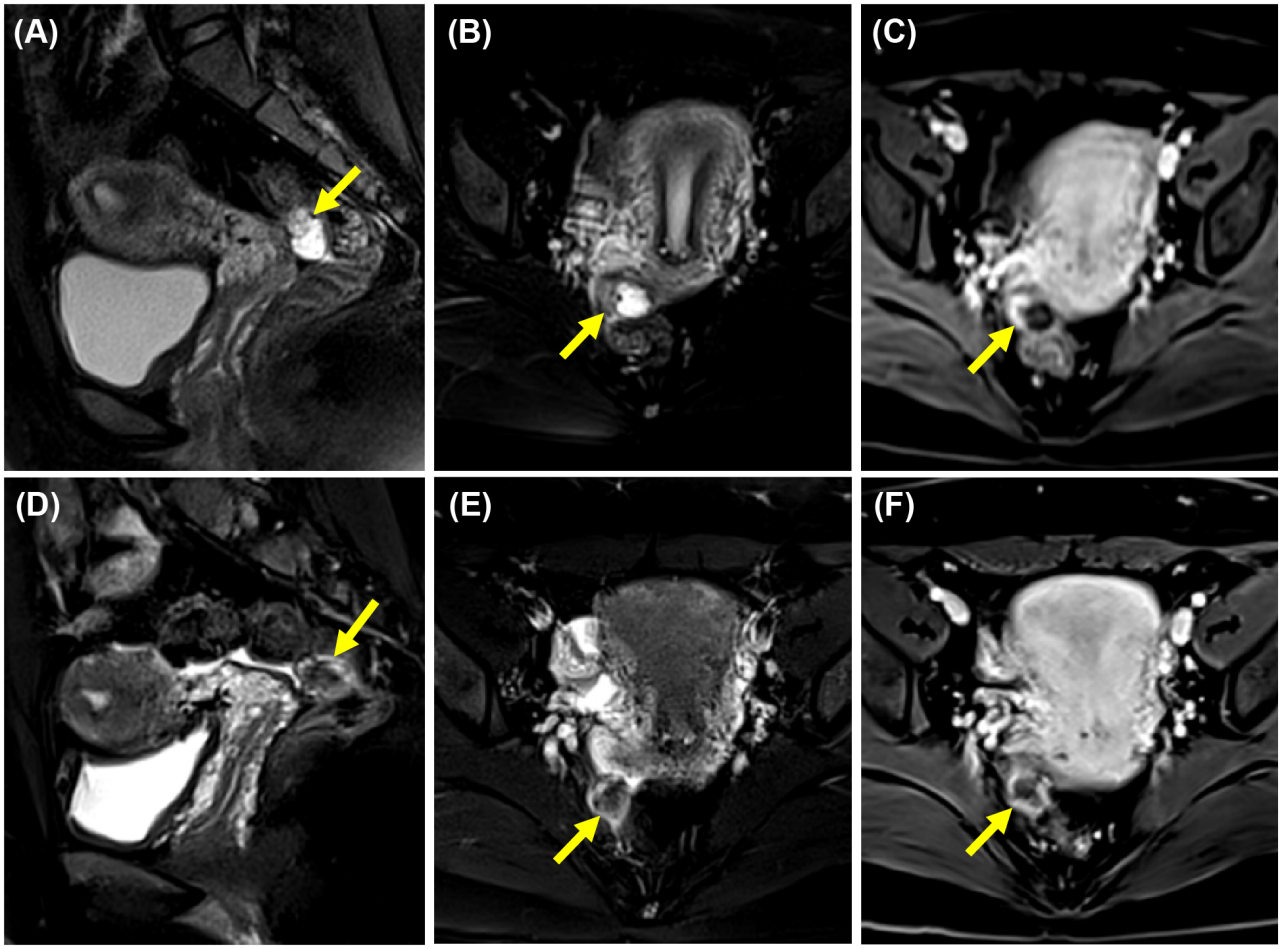
Pelvic MRI findings at the initial presentation and at recurrence. **(A–C)** Pelvic MRI in 2021 showing a right-sided cystic-solid lesion (1.7 × 1.9 × 1.9 cm) between the cervix and the rectum. On T2-weighted images **(A, B)**, the lesion shows heterogeneous high signal intensity (arrows). On the contrast-enhanced T1-weighted image **(C)**, ring-like peripheral enhancement is observed (arrow). **(D–F)** Abdominopelvic MRI in 2024 showing a round-like cystic-solid lesion (1.7 × 2.0 × 1.7 cm) at the same location. On T2-weighted images **(D, E)**, the lesion appears heterogeneous with slightly low signal intensity (arrows). On the contrast-enhanced T1-weighted image **(F)**, ring-like peripheral enhancement is again observed (arrow).

In January 2021, pelvic magnetic resonance imaging (MRI) demonstrated a 1.7 × 1.9 × 1.9 cm cystic-solid lesion between the cervix and rectum (**Figures 2A–C**). According to the FIGO 2000 system (8), the patient was classified as stage II with a prognostic score of 9. She subsequently received five cycles of etoposide, methotrexate, actinomycin D, cyclophosphamide, and vincristine (EMA-CO) chemotherapy. Each EMA-CO cycle consisted of an EMA phase with methotrexate 150/300 mg, etoposide 150 mg, and actinomycin D 0.5 mg, followed by a CO phase with vincristine 2 mg and cyclophosphamide 800 mg. All chemotherapy cycles were completed as scheduled. Adverse events included transient numbness in the fingertips and palms, Grade 1 nausea and vomiting, and Grade 2 bone marrow suppression. These symptoms were effectively managed with supportive care. Serum hCG normalized during treatment (**Figure 1A**), and complete remission was documented in April 2021.

**Figure 3:**
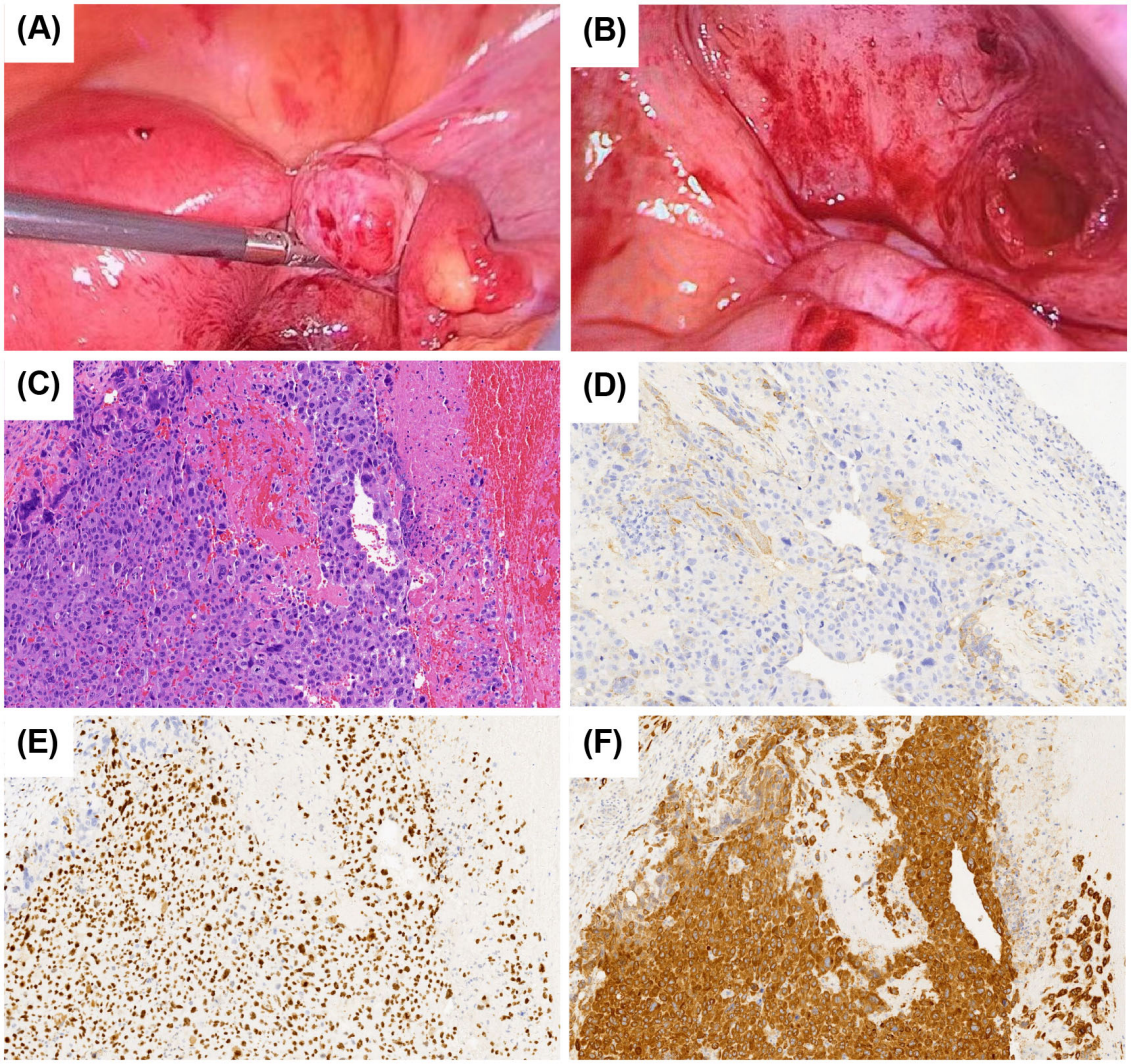
Intraoperative and histopathological features of the recurrent pelvic choriocarcinoma. **(A)** Intraoperative image of the pelvic lesion at the right uterosacral ligament. **(B)** Intraoperative image after resection, showing the defect after complete removal of the lesion. **(C)** HE staining of the resected lesion. **(D)** Immunohistochemical staining showing focal weak cytoplasmic positivity for hCG. **(E)** Ki-67 immunostaining showing a high proliferative index (~90%). **(F)** HSD3B1 immunostaining showing positivity in trophoblastic tumor cells. Panels **(C–F)** are shown at an original magnification of 10×.

**Figure 4:**
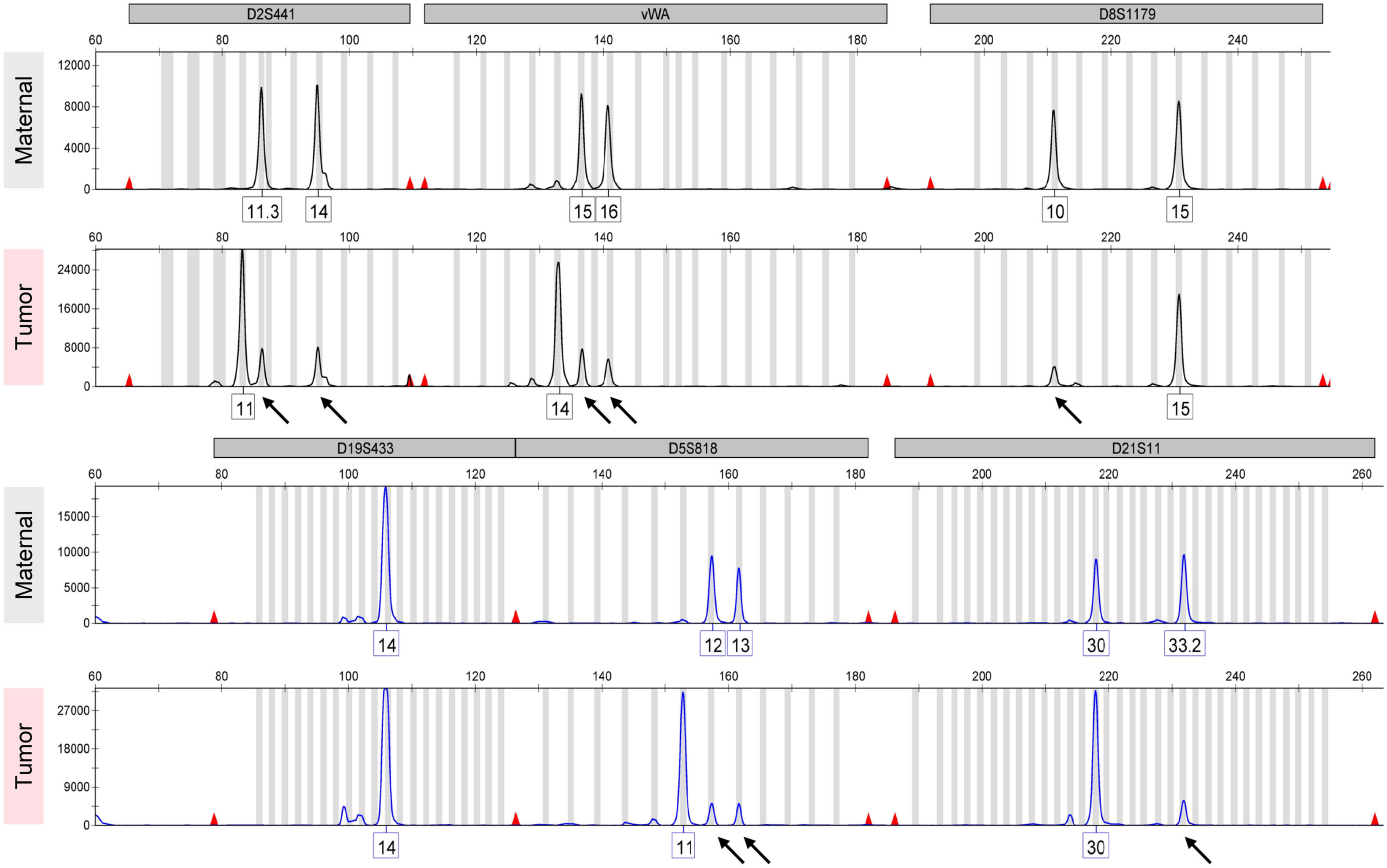
STR genotyping profiles of the recurrent choriocarcinoma tissue and the maternal reference sample. Representative electropherograms for six STR loci are shown. The upper panels represent maternal reference DNA obtained from buccal mucosa, and the lower panels represent tumor DNA from the recurrent lesion. Five loci (D2S441, vWA, D8S1179, D5S818, and D21S11) were informative, demonstrating a homozygous androgenetic diploid pattern, consistent with complete hydatidiform mole origin. D19S433 was non-informative due to homozygosity in the maternal genotype. Small peaks marked by arrows represent minor contamination from the maternal component.

After approximately 3 years of stringent contraception and undetectable hCG levels, the patient re-presented in January 2024 with hCG rising from 454 to 3,041 mIU/mL (**Figure 1B**), 1 month of amenorrhea, and scant vaginal bleeding. No obvious abnormalities of the uterus or adnexa were observed, and there was no evidence of a new intrauterine pregnancy or ectopic pregnancy. Follow-up pelvic MRI showed a 1.7 × 2.0 × 1.7 cm recurrent cystic-solid lesion at the same right uterosacral location as the primary tumor (**Figures 2D–F**), with no evidence of other disease. Given the isolated nature of the recurrence, she underwent three-dimensional laparoscopic resection, which revealed a 2.0-cm bluish-purple, hypervascular mass on the right uterosacral ligament and achieved a complete excision (**Figures 3A, B**). Pathology reconfirmed choriocarcinoma with necrosis; immunohistochemistry was positive for HSD3B1, focally positive for hCG, and a Ki-67 index of 90% (**Figures 3C–F**). A maternal buccal swab was collected at the time of recurrence as the matched reference sample. STR genotyping of the recurrent tumor demonstrated a homozygous androgenetic diploid pattern at all informative loci (**Figure 4**), indicating that the causative gestation was a CHM. On this basis, the pregnancy in 2017, clinically managed as an induced abortion, was considered the most likely antecedent pregnancy. Postoperatively, she received five cycles of TP/TE chemotherapy with paclitaxel 135 mg/m² plus cisplatin 75 mg/m² on Day 1 and paclitaxel 135 mg/m² plus etoposide 150 mg/m² on Day 15 of each cycle. After the first TE treatment cycle, the patient experienced transient leukopenia and neutropenia, followed by mild anemia. These adverse reactions responded to short-acting granulocyte colony-stimulating factor (G-CSF) injections, and the patient was then able to continue chemotherapy. During chemotherapy, serum hCG levels normalized (**Figure 1B**). She remains disease-free at 25 months of follow-up.

The overall clinical course is summarized in **Figure 1C**.

## 3 Discussion

This case represents a very rare example of pelvic GCC arising from a previously occult CHM, with recurrence at the same site after prolonged chemotherapy-induced remission. The extrauterine location and non-specific presentation initially obscured the diagnosis; indeed, the patient’s first surgery was undertaken for suspected ectopic pregnancy. This misdiagnosis is consistent with previous reports; cases of GTN presenting solely as extrauterine lesions without confirmation of an intrauterine primary lesion were often initially misdiagnosed as ectopic pregnancy, and the majority of patients required surgery to establish a definitive diagnosis (4, 9). Yamauchi et al. reported a case in which a patient underwent emergency laparotomy for suspected ruptured ectopic pregnancy, with postoperative pathology revealing choriocarcinoma. Subsequent STR analysis confirmed that the tumor originated from an occult CHM, which is very similar to our case (10).

Savage et al. reported in a case series that clinical presentation, pregnancy history, and tumor location cannot serve as reliable criteria for classifying the etiology of choriocarcinoma (11). Similarly, although a sensitive response to EMA-CO is more compatible with a GCC background (2, 12), chemosensitivity alone is insufficient to determine whether a choriocarcinoma is gestational or non-gestational. To illustrate, in a case previously reported by our group, even though the patient exhibited high sensitivity to chemotherapy, further STR genotyping classified the tumor as NGCC (13). Therefore, STR genotyping remains the most persuasive ancillary tool for distinguishing GCC from NGCC and for the etiological classification of GTN (5–7).

The STR profile of the recurrent lesion, homozygous androgenetic diploid, identified the causative gestation as a CHM. Based on her pregnancy history, the pregnancy in 2017 was considered the most likely antecedent event. However, in the absence of matched tissue from the prior gestations, it is simply the most plausible hypothesis among the clinically evident pregnancy events (the full-term vaginal delivery in 2016 and the early surgical abortion in 2017). This finding reclassified the “antecedent pregnancy” component of the FIGO prognostic score from abortion (1 point) to mole (0 points), reducing the total from 9 to 8. Although this adjustment did not alter the high-risk category or change the chemotherapy regimen in the present case, it illustrates how, in the absence of molecular genotyping, an unrecognized molar pregnancy can bias risk estimation and, when the revised total score crosses a risk-category threshold, potentially influence treatment intensity. In our patient, the “interval from index pregnancy” category was unchanged based on the available clinical history. The interval from either the full-term vaginal delivery in 2016 or the early surgical abortion in 2017 to the initial chemotherapy in January 2021 exceeded 12 months (4 points). In other clinical contexts, such reassignment may affect not only the “antecedent pregnancy” category but also the “interval from index pregnancy” category. Zhang et al. provide an example of this problem: two cases of choriocarcinoma diagnosed approximately 8 weeks after full-term delivery would have been assigned an interval score of 0 points if the recent term delivery had been regarded as the index pregnancy, but STR analysis identified their origin from prior occult molar pregnancies rather than recent full-term pregnancies (6). Gergely et al. also described a tumor whose STR profile supported CHM origin, although the exact causative pregnancy could not be definitively assigned because comparative tissue from prior gestations was unavailable (14), mirroring the circumstances of our case.

Current guidelines recommend surgery in GTN primarily for localized, chemoresistant foci (15, 16). In a case reported by Staples et al., the patient initially achieved remission following chemotherapy, but hCG levels subsequently rose again, accompanied by a persistent solitary pulmonary nodule. After undergoing wedge resection, the patient received additional Act-D therapy and ultimately achieved durable remission (17). It suggests that surgery was not used to replace chemotherapy, but rather to remove a potentially chemoresistant residual lesion, while postoperative systemic therapy remained crucial for eradicating any underlying residual disease. Similarly, in our patient, surgery was performed specifically after recurrence was confirmed to be solitary and anatomically resectable. Importantly, the second operation served both therapeutic and diagnostic purposes, achieving complete gross removal of the only visible focus of disease and providing sufficient tissue for definitive STR genotyping. Lower preoperative β-hCG has been associated with more favorable surgical outcomes in a retrospective series (18), but this should be regarded as a favorable factor rather than a prerequisite for surgery. Indeed, our patient underwent both surgeries at elevated hCG levels, the first under a misdiagnosis, the second surgery for an isolated, anatomically resectable recurrence. Therefore, the rapid postoperative biochemical remission was most likely attributable to the combined effect of complete resection and postoperative TP/TE chemotherapy. However, the independent therapeutic contribution of surgery cannot be separated from that of postoperative chemotherapy without comparison in this single case.

In conclusion, this case demonstrates that, in extrauterine choriocarcinoma with an ambiguous pregnancy history, STR genotyping of the tumor, even when obtained at recurrence, can definitively establish gestational origin, unmask an antecedent complete mole, and refine FIGO prognostic score. In this patient, however, the score correction from 9 to 8 did not alter the high-risk category or change the chemotherapy regimen. The homozygous androgenetic diploid pattern provides molecular evidence for a CHM origin of the tumor, but the absence of paired STR analysis from the antecedent pregnancy tissue precludes definitive confirmation that the tumor originated from the pregnancy in 2017, although that pregnancy remains the most likely antecedent event. Moreover, this case suggests that, in carefully selected patients with recurrent, isolated pelvic choriocarcinoma, surgical resection at elevated hCG may be justified both therapeutically and diagnostically. A limitation of this single case is that it cannot establish the general diagnostic accuracy of STR genotyping. In addition, because the patient received both surgical resection and chemotherapy, the independent therapeutic effect of surgery cannot be determined. The main value of this case is therefore to illustrate that STR genotyping may help clarify tumor origin and refine prognostic scoring, supporting its consideration in the diagnostic workup of atypical or recurrent trophoblastic tumors when sufficient tissue is available.

## Data Availability

The relevant clinical and STR genotyping results presented in this study are included in the article. The underlying raw clinical and STR genotyping data are not publicly available due to patient privacy and confidentiality restrictions. Requests to access these data should be directed to the corresponding authors and will be considered in accordance with institutional and ethical requirements.

## References

[1] Bogani G Ray-Coquard I Mutch D Vergote I Ramirez PT Prat J . Gestational choriocarcinoma. Int J Gynecol Cancer. (2023) 33:1504–14. doi: 10.1136/ijgc-2023-004704 37758451

[2] Mangla M Palo S Kanikaram P Kaur H . Non-gestational choriocarcinoma: unraveling the similarities and distinctions from its gestational counterpart. Int J Gynecol Cancer. (2024) 34:926–34. doi: 10.1136/ijgc-2023-004906 38123189

[3] Gerson RF Lee EY Gorman E . Primary extrauterine ovarian choriocarcinoma mistaken for ectopic pregnancy: sonographic imaging findings. AJR Am J Roentgenol. (2007) 189:W280–3. doi: 10.2214/AJR.05.0814 17954626

[4] Xiao P Guo T Yin R . Misdiagnosis of gestational trophoblastic neoplasia as ectopic pregnancy: a 15-year retrospective study. Front Med (Lausanne). (2022) 9:1018573. doi: 10.3389/fmed.2022.1018573 36405579 PMC9667102

[5] Buza N Hui P . Genotyping diagnosis of gestational trophoblastic disease: frontiers in precision medicine. Mod Pathol. (2021) 34:1658–72. doi: 10.1038/s41379-021-00831-9 34088998

[6] Zhang X Yan K Chen J Xie X . Using short tandem repeat analysis for choriocarcinoma diagnosis: a case series. Diagn Pathol. (2019) 14:93. doi: 10.1186/s13000-019-0866-5 31421690 PMC6698345

[7] Rozenova KA Buza N Hui P . Gestational trophoblastic disease: STR genotyping for precision diagnosis. Expert Rev Mol Diagn. (2025) 25:1–19. doi: 10.1080/14737159.2025.2453506 39801212

[8] FIGO Oncology Committee . FIGO staging for gestational trophoblastic neoplasia 2000. Int J Gynaecol Obstet. (2002) 77:285–7. doi: 10.1016/s0020-7292(02)00063-2 12065144

[9] Li J Wang Y Lu B Lu W Xie X Shen Y . Gestational trophoblastic neoplasia with extrauterine metastasis but lacked uterine primary lesions: a single center experience and literature review. BMC Cancer. (2022) 22:509. doi: 10.1186/s12885-022-09620-2 35524210 PMC9077999

[10] Yamauchi K Sato Y Usui H Sakurai A Harada R Goto M . Short tandem repeat polymorphism analysis for primary peritoneal choriocarcinoma: a case report and literature review. J Obstet Gynaecol Res. (2022) 48:2640–6. doi: 10.1111/jog.15347 35775317 PMC13469769

[11] Savage J Adams E Veras E Murphy KM Ronnett BM . Choriocarcinoma in women: analysis of a case series with genotyping. Am J Surg Pathol. (2017) 41:1593–606. doi: 10.1097/PAS.0000000000000937 28877059

[12] Wang L Wan Y Sun Y Zhang X Cheng X Wu M . Pure nongestational uterine choriocarcinoma in postmenopausal women: a case report with literature review. Cancer Biol Ther. (2019) 20:1176–82. doi: 10.1080/15384047.2019.1617564 31132027 PMC6741566

[13] Ma J Ma F Chen T Lu X Du Y Yue X . Precision diagnosis and individualized therapy of non-gestational choriocarcinoma invading the corpus uteri and cervix: a case report and literature review. Cancer Rep (Hoboken). (2025) 8:e70393. doi: 10.1002/cnr2.70393 41195577 PMC12590156

[14] Gergely L Repiska V Petrovic R Korbel M Danihel L Sufliarsky J . Short tandem repeats genotyping of gestational choriocarcinoma – our experiences. Taiwan J Obstet Gynecol. (2024) 63:73–6. doi: 10.1016/j.tjog.2023.10.004 38216273

[15] Lok C van Trommel N Braicu EI Planchamp F Berkowitz R Seckl M . Practical guidelines for the treatment of gestational trophoblastic disease: collaboration of the European Organisation for the Treatment of Trophoblastic Disease (EOTTD)–European Society of Gynaecologic Oncology (ESGO)–Gynecologic Cancer InterGroup (GCIG)–International Society for the Study of Trophoblastic Diseases (ISSTD). J Clin Oncol. (2025) 43:2119–28. doi: 10.1200/JCO-24-02326 40359461

[16] Seckl MJ Sebire NJ Fisher RA Golfier F Massuger L Sessa C . Gestational trophoblastic disease: ESMO clinical practice guidelines for diagnosis, treatment and follow-up. Ann Oncol. (2013) 24:vi39–50. doi: 10.1093/annonc/mdt345 23999759

[17] Staples JN Podwika S Duska L . The role of pulmonary resection in the management of metastatic gestational trophoblastic neoplasia: two cases of durable remission following surgery for chemo-resistant disease. Gynecol Oncol Rep. (2019) 30:100496. doi: 10.1016/j.gore.2019.100496 31693720 PMC6804952

[18] Feng F Xiang Y Li L Wan X Yang X . Clinical parameters predicting therapeutic response to surgical management in patients with chemotherapy-resistant gestational trophoblastic neoplasia. Gynecol Oncol. (2009) 113:312–5. doi: 10.1016/j.ygyno.2009.02.025 19345988

